# A *de novo* FBN1 variant likely causes congenital bilateral ectopia lentis in a crossbred horse

**DOI:** 10.1038/s41598-025-21139-7

**Published:** 2025-10-24

**Authors:** Elizabeth Esdaile, Kristopher Houston, Bradley J. Till, Roger. B. Sutton, Emma Scurrell, Max Ling, Claudia Hartley, Rebecca R. Bellone

**Affiliations:** 1 Veterinary Genetics Laboratory, University of California, Davis, Davis, CA USA; 2 The Royal (Dick) School of Veterinary Studies, The University of Edinburgh, Easter Bush Campus, Midlothian, UK; 3 Cell Physiology and Molecular Biophysics, School of Medicine, Texas Tech University Health Sciences Center, Lubbock, TX USA; 4 Cytopath Ltd, PO Box 24, Ledbury, Herefordshire UK; 5 Cheviot Vets Ltd, Pinnaclehill Industrial Estate, Kelso, UK; 6 Department of Population Health and Reproduction, School of Veterinary Medicine, University of California, Davis, Davis, CA USA

**Keywords:** Lens subluxation, Microphakia, Spherophakia, Fibrillin, Ocular disorders, Diseases, Genetics

## Abstract

Although several inherited ocular disorders have been extensively studied in horses, few reports of equine ectopia lentis exist and no genetic investigations have been reported. Ectopia lentis in humans and other species is reported to be caused by trauma, genetic variants, and systemic diseases. The most commonly reported genetic causes are dominant alleles in *FBN1*. Here we examined a 3-day old Oldenburg x Thoroughbred colt due to concerns over bilateral ocular anomalies and hypothesized that either a recessively inherited allele or a dominant *de novo* allele was the genetic cause. Examination revealed bilateral microphakia and spherophakia with medioventral lens subluxation. Histopathology of the globes was consistent with ectopia lentis. Whole genome sequencing of the affected foal was conducted, and forty-six candidate genes were evaluated for SNVs and small INDELS. Testing both hypotheses, 82 variants were identified, of which 69 were present in publicly available data from 504 horses and not investigated further. Of the 13 remaining variants, two variants were found in 3’ UTRs (*ADAMTS17* and *OAF*), ten were intronic, and one was a coding variant located in the *FBN1* gene encoding fibrillin-1 (FBN1:p.(Ala882Val)). This variant was also computationally predicted to be deleterious to protein function, including in silico modelling of FBN1 which suggests that 882Val impacts disulfide bond formation by Van der Waals clashing in a hybrid domain of the protein. The affected foal was confirmed by Sanger sequencing to be heterozygous for this variant and his clinically unaffected dam, reportedly unaffected sire, and five paternal half-siblings were homozygous for the reference allele. Additionally, the homologous human substitution is reported to be pathogenic, causing Marfan syndrome with a dominant mode of inheritance, of which ectopia lentis is a common feature. These findings support the *de novo* hypothesis with *FBN1*:p.(Ala882Val) as the likely cause of ectopia lentis in this foal, the first genetic explanation for this condition in the horse. Given the role of *FBN1* in ectopia lentis in humans and other species, *FBN1* should be evaluated as a potential candidate when other horses with this condition are identified.

**Supplementary Information:**

The online version contains supplementary material available at 10.1038/s41598-025-21139-7.

## Introduction

Ectopia lentis is characterised by a partial or full displacement of the crystalline lens due to abnormalities in the suspensory apparatus (zonules) of the lens (OMIM #129600 and #225100)^1,2^. Injury to the eye has been shown to be one cause of lens displacement in humans^3^, horses^4^, dogs^5,6^, cats^6^ and birds (especially raptors)^7,8^. Ectopia lentis has also been shown to be congenital or developmental with genetic causes identified in humans^9^, mice^10^, cattle^11,12^, pigs^13^, cats^14^, dogs^15^, and rabbits^16^. For those described with a genetic origin, other concurrent congenital conditions have also been reported. In humans, these include but are not limited to lens coloboma^9^, spherophakia (OMIM #251750)^9,17^, microphakia (OMIM #251750)^9^, glaucoma (OMIM #613086))^9,17,18^, megalocornea (OMIM #251750)^17^, Marfan syndrome (OMIM #154700)^1,2,19–21^, sulfite oxidase deficiency (OMIM *606887)^1,22,23^, Weill-Marchesani syndrome (OMIM #608990, #134797, #602091, #607511)^1,2,19,24,25^, Loeys-Dietz syndrome type 4 (OMIM #614816)^1,26^, and Traboulsi syndrome (OMIM #601552)^27^. Ectopia lentis is described as a common feature of 60% of the humans diagnosed with Marfan syndrome^20^; a connective tissue disorder also characterized by skeletal abnormalities such as long limbs as well as aortic root dilation, aneurysm, and dissection^21^.

Inherited forms of ectopia lentis have been described as simple Mendelian traits. In humans, dominant alleles in *FBN1* are by far the most commonly reported cause of Marfan syndrome^19,20^,. Variants in *ADAMTS10*^25^, *ADAMTS17*^28^, *ADAMTSL4*^29^, *ASPH*^27,30^, *CBS*^31^, *LTBPS*^17,18^, *MTHFR*^32^, *SUOX*^23^, *TGFB2*^26^, *TGFBR2*^33^ and others have also been reported to cause cases of human ectopia lentis, often as part of a systemic disease.

In comparison to the human literature, the genetics of ectopia lentis is not as well described in the veterinary literature. In cats, reports are limited to a breeding cat colony with autosomal recessive congenital glaucoma caused by a 4 bp insertion in *LTBP2* (OMIA:002017–9685)^14^. Two families of cattle with an autosomal dominant Marfan syndrome like disease were determined to be caused by to two distinct variants in *FBN1* (OMIA:000628–9913)^11,12^. The first, FBN1: p.E1200K, causes clinical symptoms most similar to neonatal Marfan Syndrome due to the severity of symptoms early in life^11^. The second, FBN1:c.8227-1G > A, is in a splicing accepter site that causes a frameshift and results in a premature termination codon 125 amino acids prior to the end of the wild type protein^12^. This variant causes a Marfan syndrome-like presentation in skeletal, ocular, and cardiovascular systems^12^. Both cattle variants are believed to have originated in the germline of mosaic, clinically unaffected bulls and have an autosomal dominant presentation^11,12^. A splice donor site variant in *ADAMTS17* causes an autosomal recessive primary lens luxation in over 20 breeds of dogs (OMIA:000588–9615)^15,34,35^, while a 6 bp deletion in *ADAMTS17* causes primary lens luxation in Chinese Shar-Pei (OMIA:000588–9615)^36^. Additionally, autosomal dominant alleles in *FBN1* cause ectopia lentis in both pig and rabbit models (OMIA:000628–9823 and 000628–9986)^13,16^. Finally, in mice a conditional knockout model of *Fbn1*(MGI:5439622)^10^ and a recessive nonsense allele in *Adamtsl4* (MGI:5902816)^37^ have been shown to cause ectopia lentis.

However, the condition has only been described in two horses and no genetic variants have been reported to cause ectopia lentis in either case. The first case was reported by Matthews & Handscombe (1983), who described a 6-month-old Arabian sport pony foal affected by bilateral cataractous and subluxated lenses^38^. Later Gerhards et al. (1992) described a case of suspected unilateral ectopia lentis in a 6-month-old Hanoverian foal^39^. However, congenital bilateral non-cataractous microphakic and spherophakic ectopia lentis has not been previously reported in the horse.

Here we identified a foal with bilateral disease and hypothesized a genetic cause was responsible for this case. Given that the dam was confirmed to be unaffected and the sire was reported by the owner to be unaffected, as well as the simple Mendelian mode of inheritance observed in other species, we hypothesized either a recessive mode of inheritance or a *de novo* dominant variant was responsible for this case. We therefore aimed to describe the ocular features of the affected foal and to perform a whole genome sequencing candidate gene prioritization approach to identify the causal variant of this phenotype.

## Methods

### Ophthalmic examination

A full ophthalmic examination of the foal and his dam was performed including neuro-ophthalmology testing, slit-lamp biomicroscopy (Kowa Optimed SL-17 Slit Lamp, Kowa Co, Japan), direct ophthalmoscopy (Keeler Professional 2.8 V, Keeler Ltd, Windsor, UK), and indirect ophthalmoscopy (Keeler Vantage Plus binocular indirect ophthalmoscope, Windsor, UK) with a 22 dioptre condensing lens (Volk Optical (Panretinal), Mentor, USA). The intraocular pressure (IOP) was also measured using rebound tonometry (TonoVet, iCare, Helsinki, Finland).

### Histopathology

Both globes were fixed in 10% neutral buffered formalin (NBF) and submitted for histopathology. Routine 4 μm haematoxylin and eosin (H&E)-stained sections of the left and right globe and lens were examined by a board-certified pathologist (ES).

### DNA extraction

DNA from whole blood was extracted from the affected colt, his dam, and five paternal half siblings using the DNA Purification from Compromised Blood Samples protocol from the Puregene Blood Kit (Cat. No. 158023, Qiagen, Germantown, MD, USA). The blood sample from the affected foal took six weeks to arrive to the laboratory after being pulled and the red cells were lysed upon arrival,, therefore, the extraction protocol was modified per the manufacturer’s suggestion by adding proteinase K to the cell lysis step for a final concentration of 100 µg/mL of proteinase K. The sample was incubated overnight at 55 °C and then processed as the remainder of the blood samples. Hair from the sire was banked as part of the parentage testing performed for the breed registry and was kindly provided by Weatherbys Scientific. DNA was extracted from the hair roots using a modified version of the crude lysis protocol described by Locke et al.^40^. The protocol was modified by increasing the number of hair roots used per horse (five to seven) and utilizing 0.5% Tween 20 and the PCR buffer and MgCl_2_ from the FastStart Taq DNA Polymerase kit (Roche Diagnostics Deutschland GmbH, Mannheim, Germany) in the extraction buffer.

### Parentage testing and breed assignment

The colt, his parents, and the five half siblings were tested by the University of California, Davis Veterinary Genetics Laboratory for 16 short tandem repeats (STRs) to confirm parentage. Parentage confirmation required zero exclusions across the 16 STRs evaluated. Breed names and VBO identification number were reported as designated for registered parents and follow those suggested by the Vertebrate Breed Ontology (VBO)^41^.

### Sequencing

Whole genome sequencing of the affected foal was completed by QB3 Genomics at the University of California, Berkeley (QB3 Genomics, UC Berkeley, Berkeley, CA, RRID: SCR_022170) in conjunction with the Center for Advanced Technology at the University of California, San Francisco. Library preparation was performed on fragmented DNA using the KAPA Hyper Prep Kit for DNA (KK8504). Libraries were evenly pooled with samples for other studies by molarity and sequenced on an Illumina NovaSeq X 150PE 10B flowcell, targeting at least 60Gb of data per sample. Raw sequencing data were converted into fastq format files using the Illumina BCL Convert software. Resulting Fastq reads were mapped to EquCab3.0 using Burrows-Wheeler Aligner (BWA) using the BWA-MEM algorithm with the option --M to mark shorter split hits as secondary^42^. Samtools v1.13 “coverage” command was used to evaluate the number of mapped reads, depth, coverage, mean baseQ, and mean mapQ from the bam file^43^. The per chromosome values were averaged across the genome. Single nucleotide variants (SNVs) and small insertion and deletions (INDELS) were identified with Freebayes v1.3.2 with the command line option –min-alternate-fraction set to 0.3^44^.

### Variant identification

A candidate gene approach was employed to prioritize variants for further evaluation. Candidate genes were identified using a literature search for genes associated with ectopia lentis in other species (Supplemental Table S1). Eighteen horses from three breeds (ten Haflingers, seven Friesians, and one Tennessee Walking Horse) who received ocular exams as part of other studies on inherited ocular disorders being conducted by the team and were not found to be affected with ectopia lentis were used as controls. These data were previously deposited at the European Variant Archive (ENA) under the following study accession numbers: PRJEB30871(six Haflingers)^45^, PRJEB36380 (seven Friesians and four Haflingers)^46^, PRJEB36381(one Tennessee Walking Horse)^47^. Two modes of inheritance were evaluated, a *de novo* dominant mode of inheritance as determined by the affected individual being heterozygous and all controls being homozygous reference for the variant, and a recessive mode of inheritance as determined by the affected individual being homozygous alternate and all controls being homozygous reference or heterozygous.

In total, 46 candidate genes were investigated for SNVs and INDELS using both a recessive and a dominant (*de novo*) model. Given the suspected rare nature of this disease, variants were prioritized first by excluding those present in a publicly available dataset with diverse breed representation (PRJEB47918)^48^. Coding variants were then prioritized for further evaluation.

The prioritized coding variant in *FBN1* was Sanger sequenced as described by Bellone et al. (2023) to confirm genotypes detected by the whole genome sequencing methodology and further test a *de novo* hypothesis using a two-primer system (FBN1_Exon22_F: 5’-CATGATGCCATTCCTGCTGT-3’; FBN1_Exon22_R: 5’-CAGTGGAATGGGCACAAGAG-3’)^49^.

### Variant modeling

PredictSNP was used to computationally evaluate the potential consequence of the coding variant found^50^. The variant form of *Equus* fibrillin-1 (XP_023473664.1) was also modelled with AlphaFold3 (AF3) and compared to a high-resolution crystal structure of the cbEGF9-hyb2-cbEGF10 fragment of human fibrillin-1 (pdb code: 2W86) to predict protein disruption^51,52^. The PDB file that includes this domain (2W86) was superimposed onto the predicted AF3 model in Pymol. Pymol displays Van der Waal clashes that occur between residues.

## Results

### History and clinical assessment

The three-day old Oldenburg (VBO_0001034) x Thoroughbred (VBO_0001083) colt and his dam, a 12-year-old Thoroughbred, were examined by the Royal (Dick) School of Veterinary Studies ophthalmology department due to concerns over bilateral ocular abnormalities identified by the primary care veterinarian. The foaling was uneventful and unassisted, and a general post-foaling clinical examination was unremarkable apart from mild angular limb deformity in all four limbs. The foal had normal mentation and was seen feeding normally from the dam, but had been witnessed to bump into a wall, raising concerns over the foal’s vision and prompting an ophthalmic examination.

**Fig. 1:**
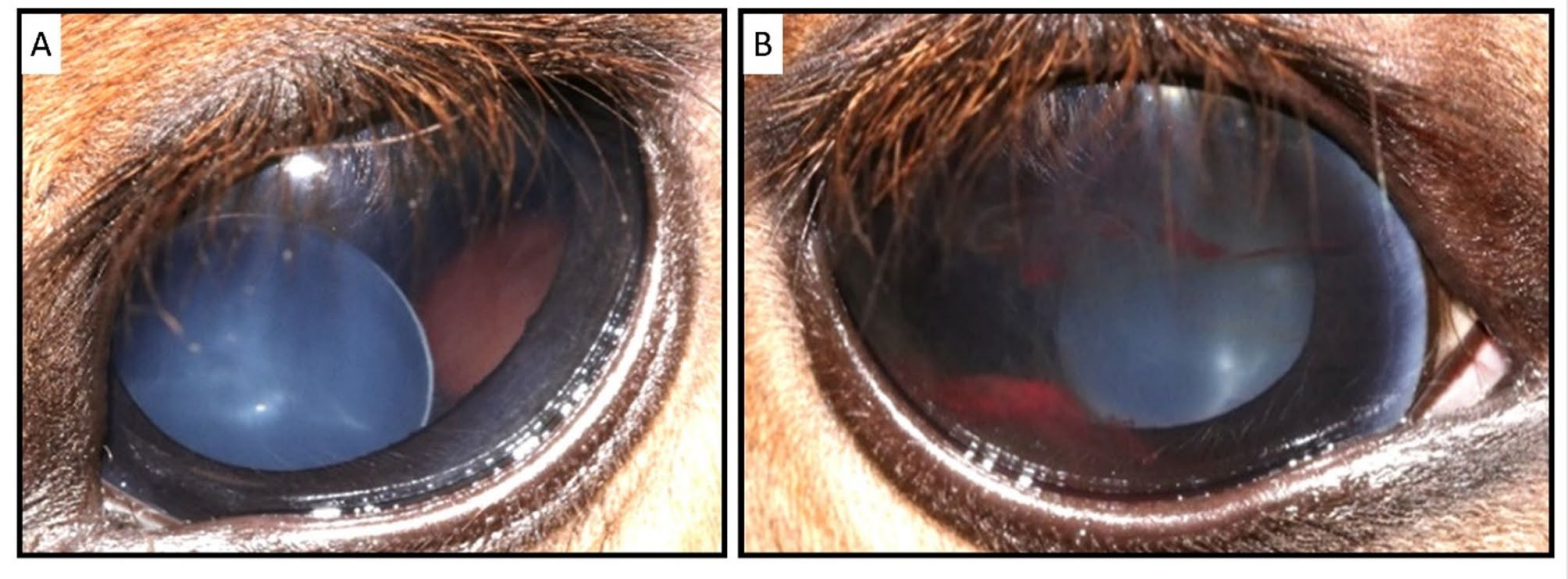
Clinical presentation showing lens subluxation in both eyes of the affected foal. Elongated zonular fibres were present in the left eye (A) on slit-lamp biomicroscopy and hyphaema was present in the right eye (B).

The intraocular pressure measurements of the colt were normal at 24mmHg bilaterally. His palpebral (lateral and medial) and dazzle reflexes were intact bilaterally, but there was an absent menace response and pupillary light reflex (PLR) in both eyes. No adnexal or corneal abnormalities were identified and there was no aqueous flare detected. Bilateral microphakia and spherophakia with medioventral subluxation was evident bilaterally, with sparse elongated zonular fibres laterally extending to the luxated lens evident in the left eye (Fig. 1A). In the right eye, there was also hyphaema, vitreal haemorrhage, and a suspected fibrovascular membrane in the anterior vitreous (Fig. 1B). Fundoscopy was challenging in the right eye but a partial retinal detachment was suspected, whereas fundoscopy of the left eye was within normal limits. The foal was subsequently euthanised using 10 ml somulose using 10 ml of Somulose, administered intravenously, due to the guarded prognosis for normal vision and an athletic career.

Ophthalmic examination of the dam was also undertaken at the same visit, and no abnormalities were noted other than a few bullet hole lesions within the non-tapetal fundus of both eyes (< 10) as an incidental finding.

The owner of the sire (an Oldenburg) reported that he had no known ocular defects, however, he was euthanized in November 2022 due to arthritis and mobility issues. He had been examined for grading in 2004 and passed a pre-purchase examination with no ocular abnormalities noted in 2009, but no specialist ocular examination was conducted before death. Additionally, ocular examinations were conducted by first opinion practitioners on five paternal-half siblings, and they were also reported to be ophthalmologically normal.

### Histopathological findings

Macroscopic examination of the colt’s globes revealed only partial adherence of the lens zonules, and focal flattening of the lens at the level of the lens equator in both globes, consistent with lens coloboma. There was mild hyphaema in the right globe.

When measured in a formalin fixed paraffin embedded (FFPE) block, the vertical axis of the left lens was 11 mm and the anterior to posterior dimension was 8 mm. Measurements were not obtained of the right lens but presumed to be similar. In both globes, there was a notch present at the lens equator (coloboma), and no cataractous changes were seen.

In both globes, there was a paucity of lens zonules, but no abnormal lens zonule protein at the level of the ciliary body epithelium was evident. There was bilateral retinal detachment, suspected to be artefactual due to the inner retinal nerve fibre layers and ganglion cell layers appearing morphologically normal, and the absence of retinal pigment epithelium (RPE) hypertrophy. However, in the right globe there was an area of segmental ventral choroidal haemorrhage with mild RPE hypertrophy suggestive of a pathological focal retinal detachment. There was no evidence of anterior segment dysgenesis or uveitis in either globe.

### Variant investigation

Whole genome sequencing yielded 2,400,927,660 mapped reads with an average depth of 18.37 and coverage of 99.65% (averaged across the chromosomes). The mean baseQ and the mean mapQ, averaged across the genome, were 35.66 and 54.98, respectively.

**Fig. 2:**
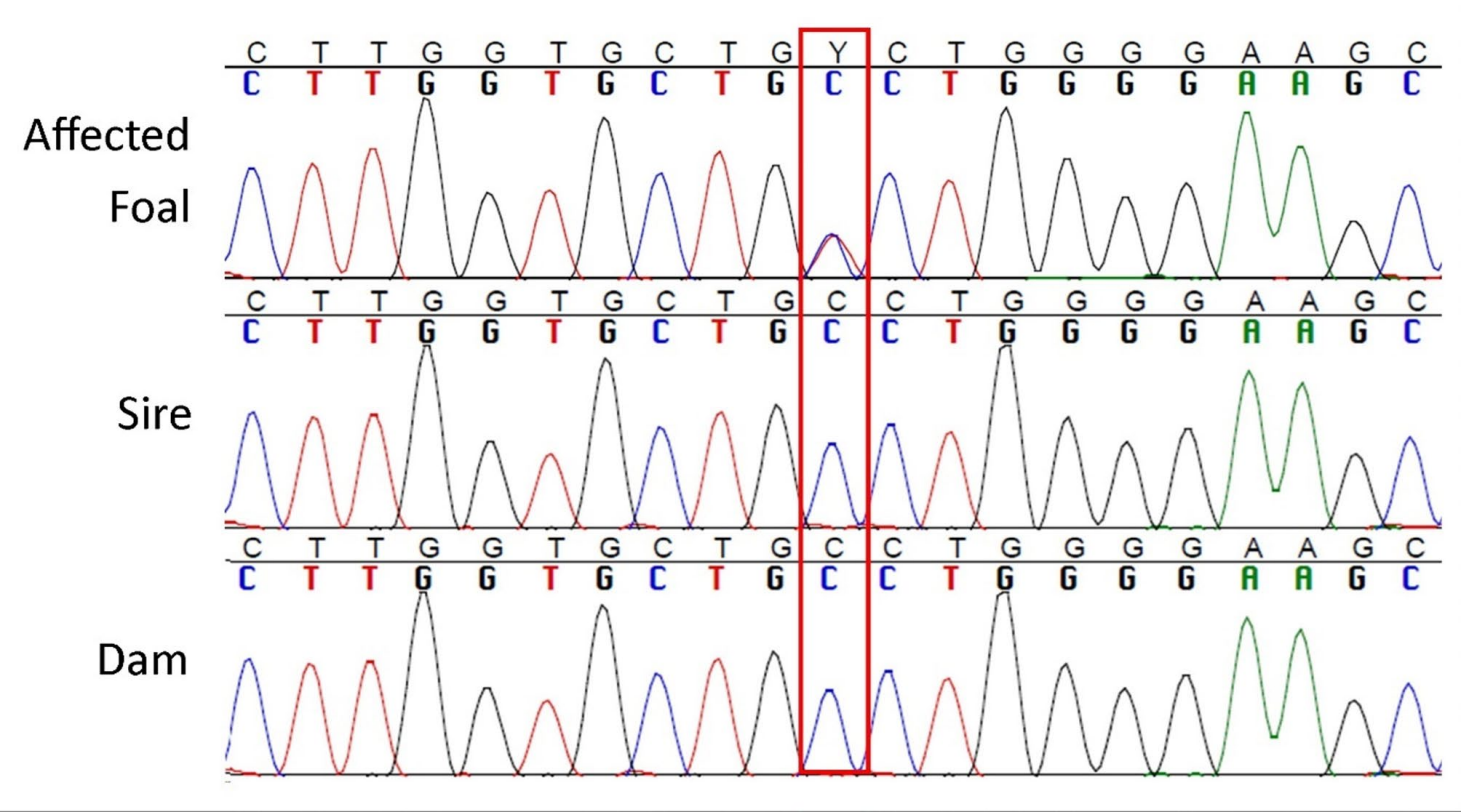
Sanger sequencing traces of the identified *de novo* coding variant in *FBN1* (NC_009144.3:g.142398596 C > T) in the affected foal and his sire and dam. The affected foal was found to be heterozygous for the identified cytosine to thymine substitution in *FBN1 (*denoted by the red box) while his sire and dam are homozygous for the reference allele, cytosine.

Eighty-two variants were identified in the candidate genes investigated. Sixty-one of these variants were heterozygous (*de novo* hypothesis) while 21 variants were homozygous (recessive hypothesis) (Supplemental Table S2). Of these 82 variants, 69 were identified in a publicly available, across breed data set of 504 horses (PRJEB47918)^48^, with 2 to 40 individuals found to be homozygous for the alternate allele for each variant identified with the recessive model, and 13 to 152 individuals found to be heterozygous for each variant identified with the dominant (*de novo*) hypothesis (Supplemental Table S2). Given that these variants were found in samples from other breeds, for a disorder that is expected to be rare in horses, they were not considered further. Of the 13 variants not identified in the publicly available data set (12 identified with a dominant *de novo* model and one identified with a recessive model), two variants were in 3’ UTRs (in *ADAMTS17*and *OAF*), ten were intronic, and one was a coding variant in *FBN1* substituting an alanine for a valine at coding position 882 (NC_009144.3:g.142398596 C > T, FBN1:p.(Ala882Val), XM_023617896.1:c.2644 C > T, XM_023617897.1:c.2644 C > T) (Table 1). The coding variant was the only variant prioritized for further investigation. Sanger sequencing confirmed the offspring to be heterozygous for the coding variant and found his dam, sire and five paternal half siblings to be homozygous reference, supporting a *de novo* hypothesis (Fig. 2).

**Table 1:** Variants identified in the affected foal that were not found in the controls or publicly available data. DbSNP reference numbers provided when available.

Model used to identify variant	Gene	Variant	Note
Recessive	*SKI*	NC_009145.3:g.47863883_47863884insA	Intronic, rs3433678633
*De novo*	*AASS*	NC_009147.3:g.78481816T > G	intronic
*De novo*	*AASS*	NC_009147.3:g.78,491,745 C > T	intronic
*De novo*	*ADAMTS10*	NC_009150.3:g.54,858,667 A > G	intronic
*De novo*	*ADAMTS17*	NC_009144.3:g.106146095G > T	3’ UTR, XM_023651354.1:r.(6273 C > T)
*De novo*	*ADAMTS17*	NC_009144.3:g.106,191,744 A > C	intronic
*De novo*	*ADAMTS17*	NC_009144.3:g.106331944G > C	intronic
*De novo*	*ADAMTS18*	NC_009146.3:g.26,552,499 A > G	intronic
*De novo*	*ADAMTS18*	NC_009146.3:g.26,552,501 C > T	intronic
*De novo*	*FBN1*	NC_009144.3:g.142,398,596 C > T	Protein coding, XP_023473664.1:p.(Ala882Val)
*De novo*	*HSPG2*	NC_009145.3:g.32902910G > T	intronic
*De novo*	*NECTIN1*	NC_009150.3:g.27,966,788 C > T	intronic
*De novo*	*OAF*	NC_009150.3:g.28,471,356 A > C	3’ UTR – XM_001501247.5:r.(1434 A > C)

### Protein modelling

**Fig. 3:**
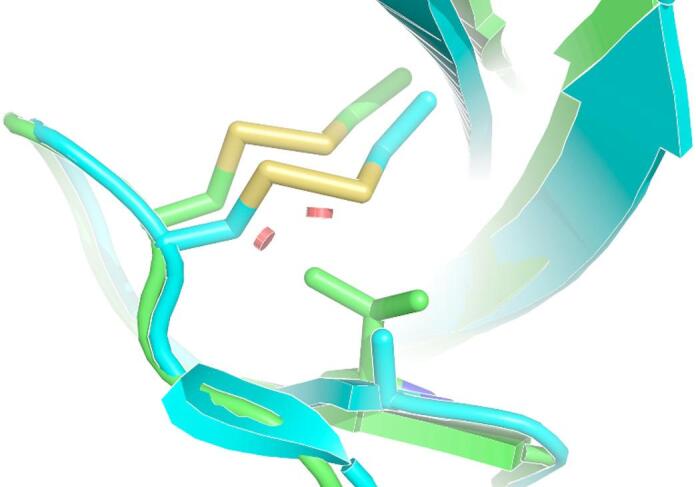
Protein modelling of human fibrillin-1 domains with equine fibrillin − 1 XP_023473664.1:p.(Ala882Val). Structural alignment of the high-resolution crystal structure of human fibrillin-1 domains cbEGF9-hyb2-cbEGF10 (pdb code: 2W86) in blue with the AlphaFold3 model of the homologous Equus fibrillin-1 domains in green (RMSD: 0.995 Å over all C-α atoms). Disulfide bonds are shown in yellow. The red octagons show the Van der Waals clashes between Val-822 and the nearby disulfide bond.

PredictSNP, a consensus classifier software, predicted FBN1:p.(Ala882Val) to be deleterious to both FBN1 isoform X1 (XP_023473664.1:p.(Ala882Val)) and isoform X2 (XP_023473665.1:p.(Ala882Val)) with normalized confidence of 61% for isoform X1 and 51% for isoform X2. Protein modelling of equine isoform 1 shows the valine substitution at residue 882 disrupts the formation of a disulfide bond in a FBN1 hybrid (hyb) domain (Fig. 3). Specifically, with modelling in Pymol, all common rotamers of the valine residue at position 882 clashed and impacted with the disulfide atoms of residues 887 and 862.

## Discussion

Ectopia lentis is characterised by displacement of the crystalline lens from its normal position in the patella fossa and can be acquired by trauma, a zonular degenerative condition (primary lens luxation), or congenital in origin^1^. In humans, ectopia lentis is well documented, with non-traumatic ectopia lentis commonly reported as part of several syndromic conditions such as Marfan syndrome (MFS) and Weill-Marchesani syndrome (WMS)^2^.

This is the first reported case of congenital bilateral ectopia lentis in the horse. Due to the age of the foal and the lack of history of trauma, a congenital form of ectopia lentis was considered. In this case, it was hypothesized that the lens vesicle failed to correctly enter the optic cup during embryogenesis. Due to early contact between the optic vesicle and the surface ectoderm being required for a normally positioned, normally shaped lens, concurrent microphakia and spherophakic are relatively common in congenital forms, as in the case reported^1,2^. The reason for absent pupillary light reflexes in both eyes was difficult to ascertain but iris hypoplasia is a possible explanation, however, this was not a reported finding in the histopathology results.

In humans, ectopia lentis results in severe visual deficits (namely diplopia and loss of accommodation and refractive error), and so treatment is indicated. Numerous surgical techniques have been described, with variable visual outcomes^53–57^. Matthews et al. (1983) described the surgical treatment of a 6-month-old foal with bilateral cataracts and lens subluxations using extracapsular lens extraction (ECLE) in one eye, and intracapsular lens extraction (ICLE) in the other^38^. However, long-term follow-up was not available. A more recent retrospective study investigating visual outcomes of surgical therapy for lens luxation/subluxation in horses reported a high rate of intra- and post- operative complications resulting in poor visual outcomes in the majority (80%) of cases^57^. In light of this, the foal studied here was euthanized due to the severity of the clinical findings and suspected visual deficits rendering the foal unfit for the purpose for which it was bred.

*FBN1* is a well-studied gene in humans with over 803 variants known to cause Marfan syndrome and related fibrillopathies, including ectopia lentis^19^ and a total of 2,361 variants in *FBN1* are reported to be pathogenic/likely pathogenic^58^. *FBN1* encodes a large macromolecule that forms microfibrils that contribute to the function and integrity of connective tissue including the ciliary zonule. Protein modelling in this study predicted that p.(Ala882Val) disrupts the formation of disulfide bond of the fibrillin-1 hyb domains, these disulfide bondswere previously shown to play a role in structural integrity of the microfibrils^52^. Given the role of FBN1 in fibrillogenesis, the link of variants in this gene in human and cattle cases of ectopia lentis, and the computationally predicted functional impact of the variant identified here, FBN1:p.(Ala882Val), is likely causal for this equine case.

While this study provides strong evidence for FBN1:p.(Ala882Val) as the likely cause in this case, the candidate gene approach and variant detection method employed do not allow us to rule out variants in other genes not investigated or structural variants as the cause. Nonetheless, the same variant identified here has also been reported in humans with Marfan Syndrome with ectopia lentis and is predicted by ClinVar to be pathogenic or likely pathogenic (Accession: VCV000200001.36) (Supplemental Table S3). At least 14 affected people, 12 of which are unrelated, have been reported in the published literature to have this variant, with more reported in ClinVar (Supplemental Table S3)^59–69^. Of these 14 individuals, diagnoses varied from Marfan syndrome to aortic aneurism to unspecified fibrillinopathy (Supplemental Table S3).

A second substitution of the same amino acid, FBN1:p.(Ala882Thr), has also been identified in humans^70^. This substitution is interpreted by ClinVar (Accession: VCV000853703.10) to be pathogenic and is present in patients with Marfan syndrome and familial thoracic aortic aneurysm and dissection, thus providing further support of the importance of alanine at amino acid 882 for normal protein function.

The identification of the FBN1:p.(Ala882Val) in this foal with ectopia lentis, along with the sire and dam having no reported ocular abnormalities, and the absence of this allele in the parents and paternal half siblings suggests that this variant resulted from a *de novo* mutation in the germline of one of the parents or during early embryonic development of the foal, similar to most *FBN1* variants in humans. Although major skeletal findings were not identified, it is not possible for Marfan-like syndrome or other fibrillopathies to be ruled out as the foal was not examined for skeletal abnormalities beyond the mild angular limb deformities reported, or for aortic dissection and rupture, and a necropsy was not conducted. Limitation of clinical phenotyping in this case is also compounded by the need to evaluate individuals into adulthood as some symptoms may not appear until then. The foal studied here was euthanized due to the severity of the clinical findings that rendered the foal unfit for the purpose for which it was bred and therefore further follow-up in this case is not possible.

However, it seems likely, given the reported mild limb angle deformities and the confirmed ectopia lentis, this case could be considered the first Marfan-like equine case with a genetic origin identified. Additional functional work is needed to investigate the hypothesis that FBN1:p.(Ala882Val) disrupts proper disulphide bond formation impacting fibrillin organization. This study supports further investigation of *FBN1* variants in the horse, to determine if there are other examples of Marfan-like syndrome with variable presentations in the horse. Given the number of deleterious *FBN1* variants reported in humans, more work is needed to identify and investigate *FBN1* variants in horses and the potential phenotypic consequences.

## Conclusion

We detected a *de novo* variant, FBN1:p.(Ala882Val), to be likely causal to bilateral ectopia lentis in a three-day old foal. This is the first report of a horse with bilateral ectopia lentis for which a potential genetic mechanism was unveiled. Given the number of pathogenetic *FBN1* variants in humans, it is surprising that this is the only reported *FBN1* variant connected to a phenotype in the horse.

## Supplementary Information

Below is the link to the electronic supplementary material.

Supplementary Material 1

## Data Availability

Whole genome sequencing data from the affected foal has been deposited in the European Nucleotide Archive under project accession PRJEB75138.
